# A Comparative Study of the Impact of Theta-Burst and High-Frequency Stimulation on Memory Performance

**DOI:** 10.3389/fnhum.2016.00019

**Published:** 2016-02-03

**Authors:** Yating Zhu, Rubin Wang, Yihong Wang

**Affiliations:** Department of Science, Institute for Cognitive Neurodynamics, East China University of Science and TechnologyShanghai, China

**Keywords:** working memory, long-term memory, HFS, TBS, LTP

## Abstract

The transformation of the information stored in the working memory into the system of long-term memory depends on the physiological mechanism, long-term potential (LTP). In a large number of experimental studies, theta-burst stimulation (TBS) and high-frequency stimulation (HFS) are LTP induction protocols. However, they have not been adapted to the model related to memory. In this paper, the improved Camperi–Wang (C–W) model with Ca^2+^ subsystem-induced bi-stability was adopted, and TBS and HFS were simulated to act as the initial stimuli of this working memory model. Evaluating the influence of stimuli properties (cycle, amplitude, duty ration) on memory mechanism of the model, it is found that both TBS and HFS can be adopted to activate working memory model and produce long-term memory. Moreover, the different impacts of two types of stimuli on the formation of long-term memory were analyzed as well. Thus, the importance of this study lies firstly in describing the link and interaction between working memory and long-term memory from the quantitative view, which provides a theoretical basis for the study of neural dynamics mechanism of long-term memory formation in the future.

## Introduction

Memory is an advanced cognitive function of the brain as well as a basic step in human thinking, thus becoming an important research topic in neural information processing. Hermann Ebbinghaus, who first studied memory, indicated that stable memory can be achieved through the duplication of the memory object (Ebbinghaus, 1913). Afterwards, neuroscientists classified the memory according to the memory properties. There are three memory systems widely studied and these include long-term memory, short-term memory, and working memory.

Researchers from different perspectives have given different ideas on the relationship between short-term memory and working memory. Baddeley et al. supposed that working memory is a system with multiple components, which can store and process the information in short-term memory (Baddeley et al., 1975); Engle et al. proposed that working memory refers to attention-related parts of short-term memory (Engle, 2002). Many studies have focused on working memory (Daneman and Carpenter, 1980; Conway et al., 2001; Kane et al., 2001; Colliaux et al., 2009), because working memory has been found to correlate with intellectual aptitudes better than short-term memory as well as any other particular psychological process. For example: Daneman and Merikle found that combining the information stored and processed by working memory can predict language comprehension well (Cowan, 2008). Cowan believed that working memory has been defined in three different ways: as short-term memory applied to cognitive tasks, as a multi-component system that holds and manipulates information in short-term memory, and as the use of attention to manage short-term memory. That is to say, working memory includes short-term memory and other processing mechanisms that help to make use of short-term memory (Daneman and Merikle, 1996). Based on the perspectives of different researchers and the comparison of the functional characteristics of short-term memory and working memory, it is more helpful to study the mechanism of memory formation with working memory.

The differences between long-term memory and short-term memory are as follows: (1) short-term memory demonstrates temporal decay and chunk capacity limits (Tarnow, 2010); (2) long-term memory demonstrates memory time for more than a minute or even a lifetime without capacity limits (Daneman and Carpenter, 1980). Cowan pointed out the relationship between short-term memory and long-term memory. He considered that short-term memory is derived from a temporarily activated subset of information in long-term memory, and if the activated subset is not refreshed, it may decay over time (Cowan, 1988, 1995, 1999, 2001). In other words, if the information stored in short-term memory is further activated, it may form long-term memory (Tarnow, 2009).

Based on what have been discussed in relation to long-term memory, short-term memory and working memory, we consider that the information held in working memory can be transformed into long-term memory after rehearsal, coding, and linking to individual experience. The formation mechanism of long-term memory is long-term potentiation (LTP). Cooke et al. proposed that LTP and long-term memory have a lot in common. For example, both can be rapidly induced, both depend on the synthesis of new proteins and both can last for several months. Based on these factors, LTP can be the best candidate to study cell mechanism (Cooke and Bliss, 2006; Leleu and Aihara, 2012).

Stimuli with different properties possess different functions (Haab et al., 2011; Ishino and Sakurai, 2014). In so many experiments, theta-burst stimulation (TBS) and high-frequency stimulation (HFS) are LTP induction protocols (Otto et al., 1991; Yun et al., 2002; Sweet et al., 2014). TBS is a repeating pattern of short burst of pulses (e.g., 4 pulses at 100 Hz) with brief pauses (~200 ms) between bursts (Perez et al., 1999). HFS is a repeating pattern consisting of a 1 s train of pulses delivered at 100 Hz, which is often repeated with intervals up to several seconds long (Yun et al., 2002). Previous researches have shown that magnitude of LTP is affected by some factors such as frequency, number of pulses, and stimulus intensity. Hernandez et al. compared the impact of TBS and HBS with the same pulse number on LTP in the system, and studied the impact of the increase in pulse number on LTP (Hernandeza et al., 2005).

In this paper, our studies are based on C–W model with Ca^2+^ subsystem-induced bi-stability (Fall and Rinzel, 2006). The C–W model is proposed by Camperi and Wang (1998), aiming to model and simulate the working memory activity during memory-guided oculomotor delayed-response experiment by Funahashi et al. (1989). In C–W model, bi-stability is controlled by the current equation and parameters. After that, Fall and Rinzel replace the intrinsic conditional bi-stability in C–W model with an intracellular Ca^2+^ subsystem which is of physiological significance. The simulation results from C–W model with Ca^2+^ subsystem-induced bi-stability are found to be in good agreement with the experimental results from the study of working memory by Funahashi et al. Based on C–W model with Ca^2+^ subsystem-induced bi-stability, we adjusted the initial stimulus current in the model into the forms of TBS and HFS, and assessed the impacts of these two kinds of initial stimulus current on memory mechanism of the model by control variate method. Furthermore, TBS and HFS were applied to the model for inducing long-term memory, and the effects of long-term memory induced by the two kinds of stimuli protocols with the identical pulse number were compared. The research reveals how to form long-term memory with two kinds of LTP induction protocols on the basis of working memory, thus providing the basis for the study of the formation mechanisms of long-term memory.

## Models and methods

We adopted the use of C–W model with Ca^2+^ subsystem-induced bi-stability (Funahashi et al., 1989) and the improvements of firing-rate network proposed by Liang et al. (2010). Liang et al. substituted the linear function of the firing rate in traditional firing-rate network model for the non-linear cubic function of the firing rate, which simplified the model, maintaining the simulation effects. Therefore, this model was adopted to study the impacts of the characteristics of initial stimulus current on the memory mechanism of the model.

The network consists of N neurons, each labeled by its preferred cue location or memory field θ_*i*_ with range from −π to π. In this limit, the equation of the firing rate *r*(θ_*i*_, *t*) is:

(1) $$\begin{array}{l} \tau_{r}\frac{d}{dt}r(\theta_{i},t)=-f[r(\theta_{i},t)]+g[I(\theta_{i},t)]\\ \;\begin{array}{l} f(r)=r \end{array} \end{array}$$

where τ_*r*_ is the time constant. The function *f*(*r*) and *g*(*I*) stand for the intrinsic properties of the neuron. By setting *dr*∕*dt* = 0, the steady state of this system can be obtained, thereby the neuronal input-output relation is *r* = *g*(*I*). The piece-wise linear function form of *g*(*I*) is as follows:

(2) $$g(I)\{\begin{array}{cc} 0 & \;I<0\\ 0.2 & 0\leq I<1\\ 5I-4.8 & 1\leq I<2\\ 0.8I+3.6 & \;2\leq I \end{array}$$

The total input to each neuron *I* consists of an external input *I*_*ext*_ for the transient cue and a recurrent synaptic input *I*_*syn*_. Considering that Ca^2+^ release might increase the efficacy of synaptic input; therefore the input *I* is represented as:

(3) $$\begin{array}{l} I\left(\theta_{i},t\right)=I_{ext}\left(\theta_{i},t\right)+\left(1+N_{Ca}\right)I_{syn}\left(\theta_{i},t\right) \end{array}$$

where *N*_*Ca*_ represents intracellular Ca^2+^ concentration. The external input *I*_*ext*_ contains a constant bias input *I*_0_ and a cue stimulus:

(4) $$\begin{array}{l} I_{ext}\left(\theta_{i},t\right)=I_{0}+I_{cue}{\left(\frac{1+\cos\left(\theta_{i}-\theta_{0}\right)}{2}\right)}^{p} \end{array}$$

The variable θ_0_ represents the cue position and *p*controls the width of cue stimulus. Constant *I*_*cue*_ was taken as 1 during 1–1.5 s, but as 0 in the rest of the time range in the previous work. Here, we define *I*_*cue*_ as the forms of HFS and TBS, which are simulated with the cycle of the square signal. In this work, function “square” in Matlab was used to make the square signal with a specific frequency, amplitude, and duty ration. Specifically, the initial HFS was simulated as follows: The frequency of square signal was set as 130 Hz. The range of amplitude was 0–1; The duty ration was 20%; The sustaining 1-s-activity with the starting time at 1 s was defined as one cycle (consisting of 130 pulses). The initial TBS was simulated as follows: The frequency of square signal was set as 200 Hz; The range of the amplitude was 0–1 and the duty ration was 20%; It was sustained for 0.05 s with the starting time at 1 s (consisting of 10 pulses) and the interval period of 1.05–1.2 s (the signal value was 0 during interval period). The activity of 1–1.2 s was defined as a cycle and then repeated for five cycles (consisting of 50 pulses).

The synaptic input *I*_*syn*_ is given by convolution over the weights and firing rates:

(5) $$\begin{array}{l} I_{\;syn}\left(\theta_{i},t\right)=\;\sum_{j=1}^{N}\frac{1}{\;N}\left[W\left(\theta_{i}-\theta_{j}\right)+\Delta W\right]r\left(\theta_{j},t\right) \end{array}$$

The static connectivity weight *W* between two neurons has an inhibition form which is given by:

(6) $$\begin{array}{l} W\left(\theta \right)=-W_{I}+W_{E}{\left(\frac{1+\cos\theta }{2}\right)}^{q} \end{array}$$

where constants *W*_*I*_ and *W*_*E*_ represent the strengths of the inhibitory and excitatory interactions, respectively. Δ*W* is the amendment of the synaptic weights. It is given by:

(7) $$\begin{array}{l} \frac{d\Delta W}{\;dt}=-\frac{\Delta W}{\tau_{w}}+kr \end{array}$$

Here, τ_*w*_ is the time constant and *k* is the proportional coefficient.

The dynamic function of Ca^2+^ subsystem mainly depends on the level of the second messenger inositol 1,4,5 trisphosphate (IP3), in which *N*_*IP*3_ represents IP3 concentration. Thus, a balanced equation for the evolution of Ca^2+^ concentration includes release of Ca^2+^ concentration through the IP3R (IP3 sensitive Ca^2+^ channel) into the cytosol (*J*_*IP*3*R*_), removal of Ca^2+^ concentration induced by the reuptake of SERCA pumps from the cytosol (*J*_*SERPM*_), a leak flux concentration from the endoplasmic reticulum (ER) to the cytosol (*J*_*Leak*_) and an important contribution to Ca^2+^ influx concentration from network activity (*J*_*syn*_).

$$\begin{array}{lll} \frac{dN_{Ca}}{dt} & = & J_{IP3R}-J_{SERPM}+J_{Leak}+J_{syn}\\ J_{IP3R} & = & V_{IP3R}m_{\infty }^{3}h_{3}\left(N_{CaER}-N_{Ca}\right)\\ J_{SERPM} & = & V_{SERPM}\frac{N_{Ca}^{2}}{k_{SERPM}^{2}+N_{Ca}^{2}}\\ J_{Leak} & = & V_{Leak}\left(N_{CaER}-N_{Ca}\right)\\ \frac{dh}{dt} & = & \frac{h_{\infty }-h}{\tau_{h}},h_{\infty }=\frac{k_{inh}}{k_{inh}+N_{Ca}}\\ m_{\infty } & = & \frac{N_{IP3}}{N_{IP3}+k_{IP3}}\frac{N_{Ca}}{N_{Ca}+k_{act}}\\ J_{syn} & = & \alpha g\left(I_{1}\right) \end{array}$$

(8) $$\begin{array}{l} I_{1}\left(\theta ,t\right)=I_{ext}\left(\theta ,t\right)+I_{syn}\left(\theta ,t\right) \end{array}$$

where *h* represents the proportion of IP3R's not inactivated by *N*_*Ca*_, and *N*_*CaER*_ represents the Ca^2+^ concentration through ER. The gating relation *m*_∞_ is the fast activation and α is the proportional coefficient.

In this paper, we simulated the process of transformation from working memory to long-term memory using Matlab (R2011b) with a time step of 0.001 s. The number of neurons was 128. The parameter value, *IP*3 = 0.562, which ensured the bi-stability of Ca^2+^ subsystem was used. Unless otherwise indicated, other parameter values were identical to those used in the model by Liang et al. (2010), which are shown in Table 1.

**Table 1:** **Model parameters**.

**C–W model**		**Ca**^**2+**^ **subsystem**	
**Parameter**	**Values**	**Parameter**	**Values**
Integration time (*τ_*r*_*)	0.1	ER Ca^2+^ concentration (*N_*CaER*_*)	11
Stimulus shape exponent (*p)*	1	IP3R Ca^2+^ flux (*V_*IP*3*R*_*)	80
Weight shape exponent (*q)*	1	Ca^2+^ leak flux (*V_*Leak*_*)	0.00032
Background activity (*I_0_)*	0.2	Ca^2+^ pump flux (*V_*SERPM*_*)	3.33
Inhibitory strength (*W_*I*_*)	2	Ca^2+^ pump sensitivity (*k_*SERPM*_*)	0.4
Excitatory strength (*W_*E*_*)	2.8	IP3R IP3 sensitivity (k_*IP*3_)	0.4
Cue position (*θ_0_*)	0	IP3R Ca^2+^ inhibition constant (*k_*inh*_)*	1.4
Dynamic synaptic time constants (*τ_*r*_*)	0.02	IP3R Ca^2+^ activation constant *(k*_*act*_*)*	1.1
Dynamic synaptic proportional coefficient (*k)*	0.1	IP3R in act, time constant (*τ_*h*_*)	0.1
		Coupling parameter (α)	0.7

We investigated the effect of the properties (cycle, amplitude, and duty ration) of the two types of stimulus (TBS and HFS) on memory performance using control variate method, in which the duration of each pulse was proportional to duty ration of stimulus; stimulation time was proportional to cycle and stimulus intensity was proportional to amplitude. Moreover, the frequencies of these two kinds of stimulus were set to meet the form of TBS and HFS. Thus, the stimuli can be studied as potential mechanisms for the formation of long-term memory. We hoped to transform working memory into long-term memory in the model by adjusting the comprehensive effect of stimulus (TBS, HFS) properties, so as to further analyze the impact of the properties of the two kinds of stimuli on long-term memory and compare their differences.

## Results

**Figure 1:**
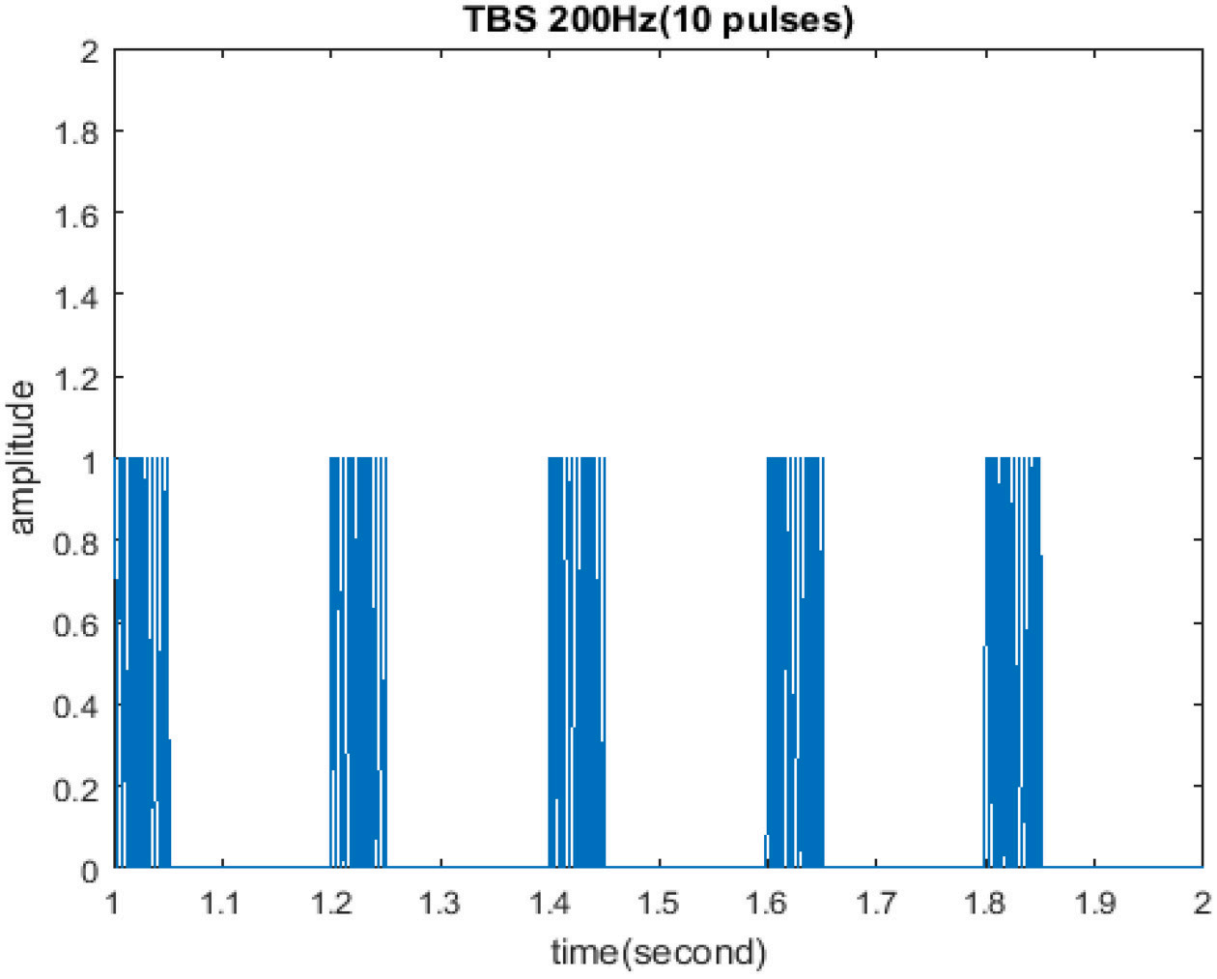
**Initial form of TBS**. TBS consisted of ten pulses at 200 Hz repeated with 200 ms inter-burst-intervals in which cycle was 5, amplitude was 1, and duty ration was 50%.

The initial form of TBS studied in this paper is shown in Figure 1. It consisted of ten pulses at 200 Hz repeated with 200 ms inter-burst-intervals in which cycle was 5, amplitude was 1, and duty ration was 50%. Some parameters remained unchanged including: stimulus frequency 200 Hz; pulse number of each cycle 10; cycle interval 200 ms. However; cycle, amplitude, and duty ration were adjusted according to the control variate method, so as to evaluate the impact of these three stimuli properties on the memory model.

**Figure 2:**
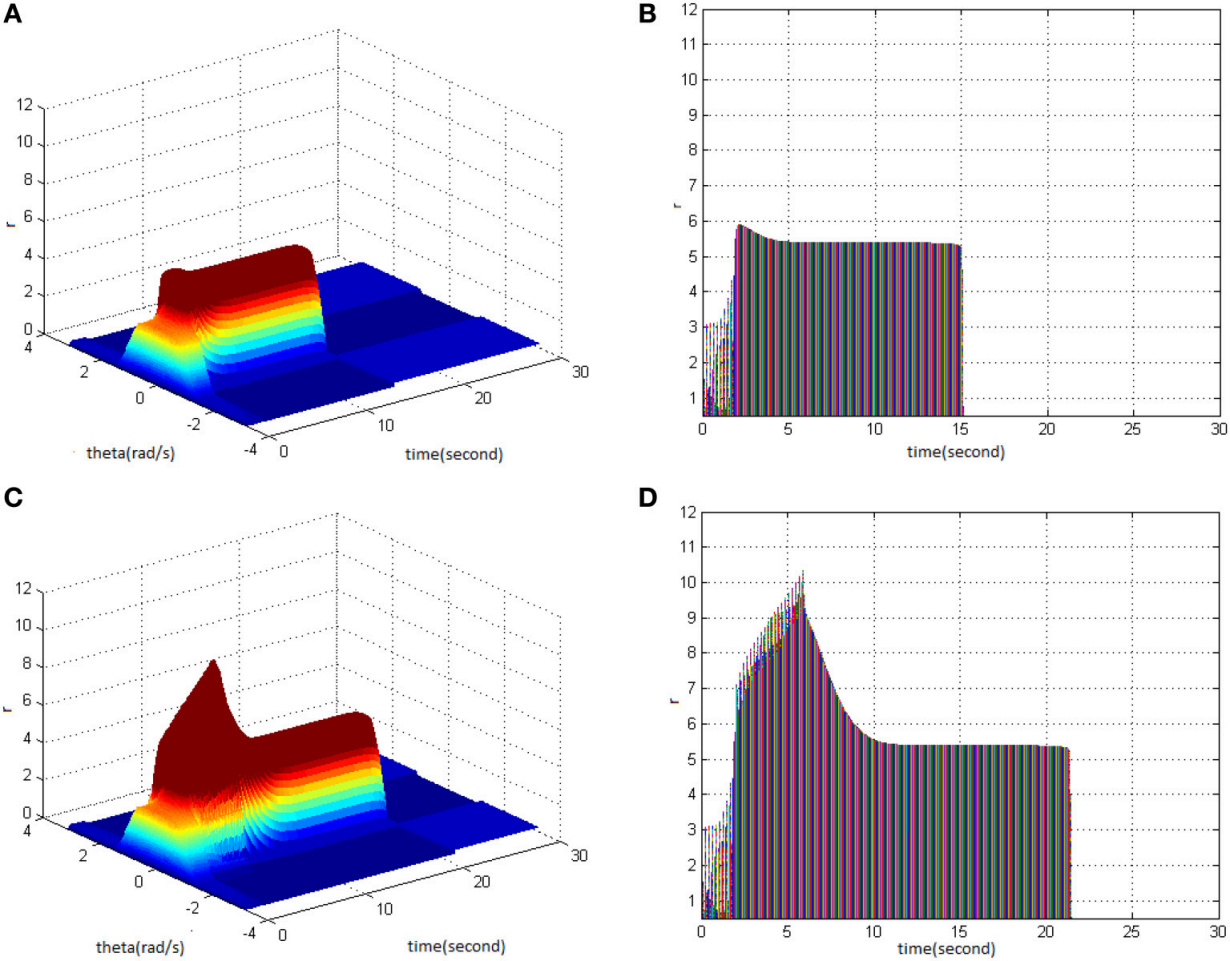
**Effect of TBS cycle on memory performance. (A)** Space-time plots of the firing activity with amplitude 1.8, duty ration 50%, and cycle 10. **(B)** The projection of **(A)** in plane time-*r*. **(C)** Space-time plots of the firing activity with amplitude 1.8, duty ration 50%, and cycle 30. **(D)** The projection of **(C)** in plane time-*r*.

**Figure 3:**
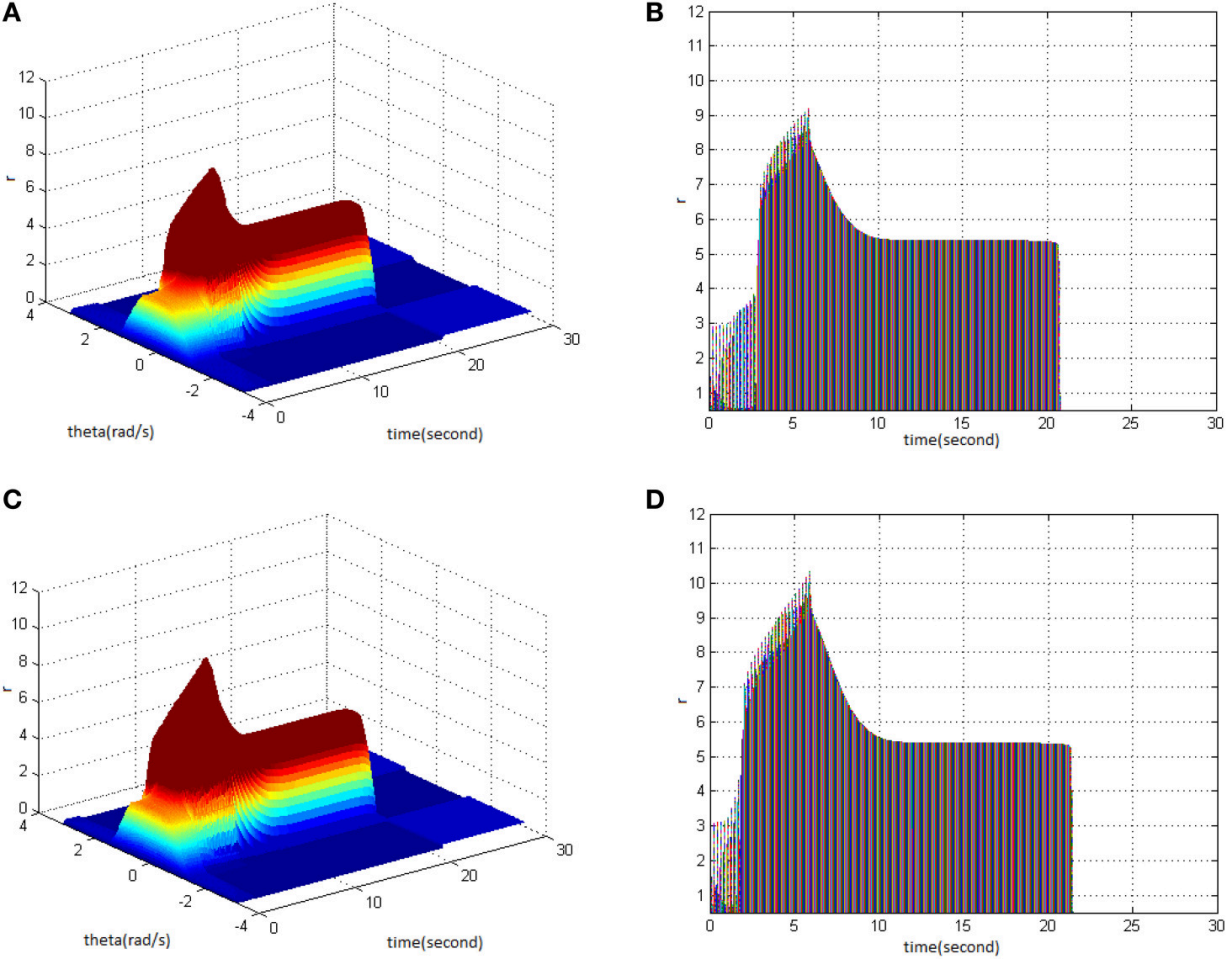
**Effect of TBS amplitude on memory performance. (A)** Space-time plots of the firing activity with cycle 30, duty ration 50%, and amplitude 1.6. **(B)** The projection of **(A)** in plane time-*r*. **(C)** Space-time plots of the firing activity with cycle 30, duty ration 50%, and amplitude 1.8. **(D)** The projection of **(C)** in plane time-*r*.

**Figure 4:**
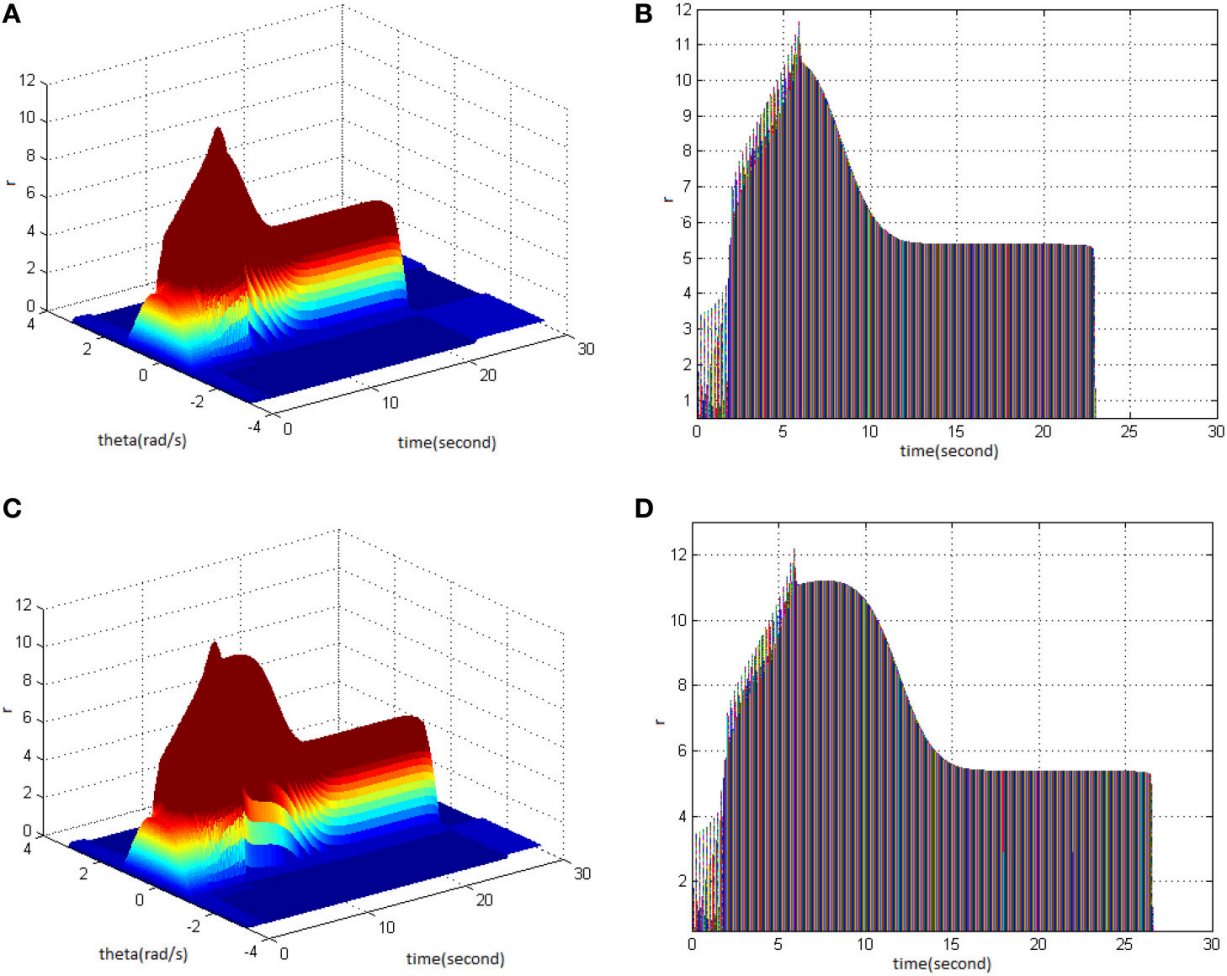
**Effect of TBS duty ration on memory performance. (A)** Space-time plots of the firing activity with TBS properties of cycle 30 amplitude 1.4, and duty ration 65% **(B)** The projection of **(A)** in plane time-*r*. **(C)** Space-time plots of the firing activity with TBS properties of cycle 30, amplitude 1.4, and duty ration 80%. **(D)** The projection of **(C)** in plane time-*r*.

**Figure 5:**
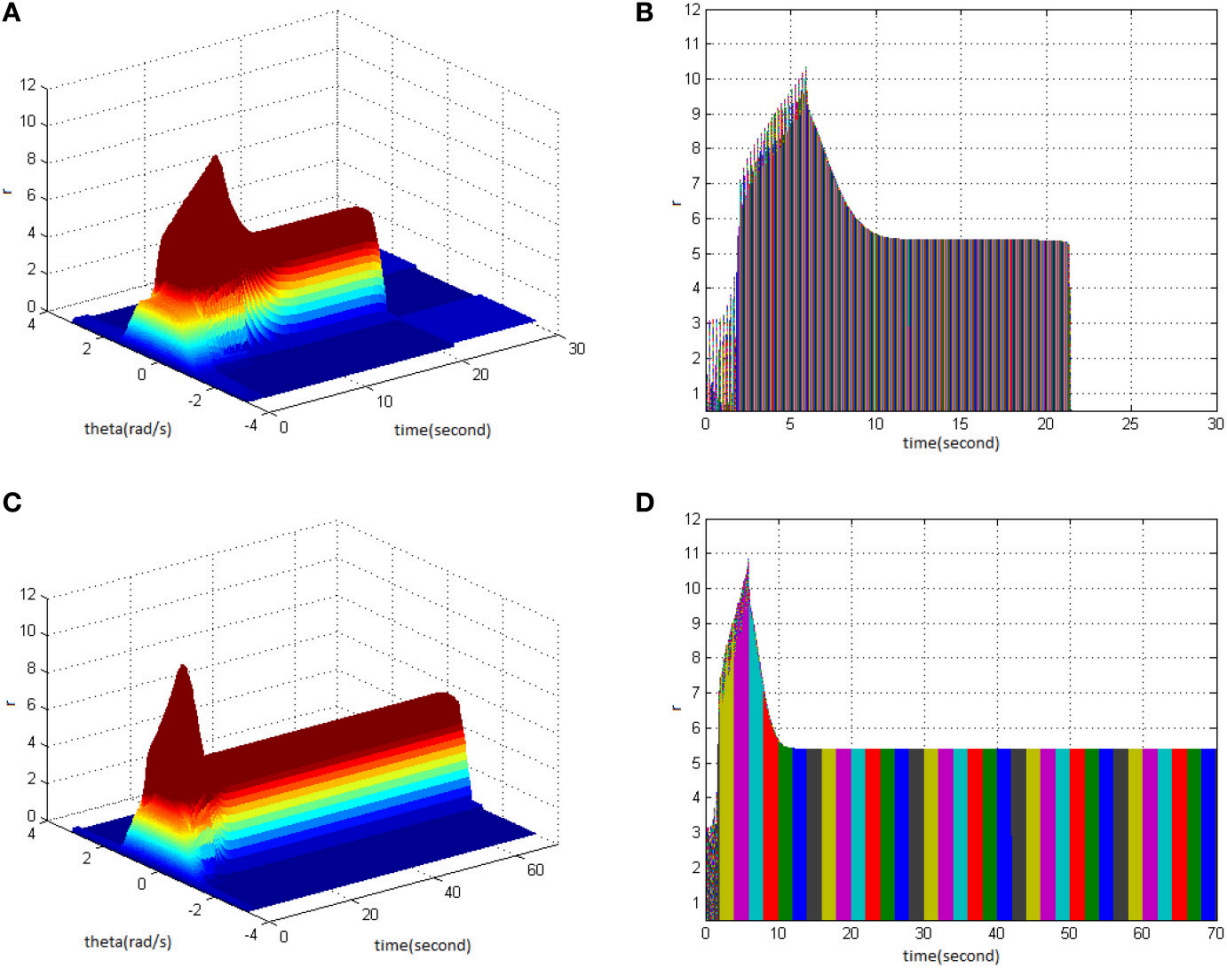
**Critical value of TBS properties to produce long-term memory**. **(A)** Space-time plots of the firing activity with TBS properties of cycle 30, amplitude 1.8, and duty ration 50%. **(B)** The projection of **(A)** in plane time-*r*. **(C)** Space-time plots of the firing activity with TBS properties of cycle 30, amplitude 1.9, and duty ration 50%. **(D)** The projection of **(C)** in plane time-*r*.

**Figure 6:**
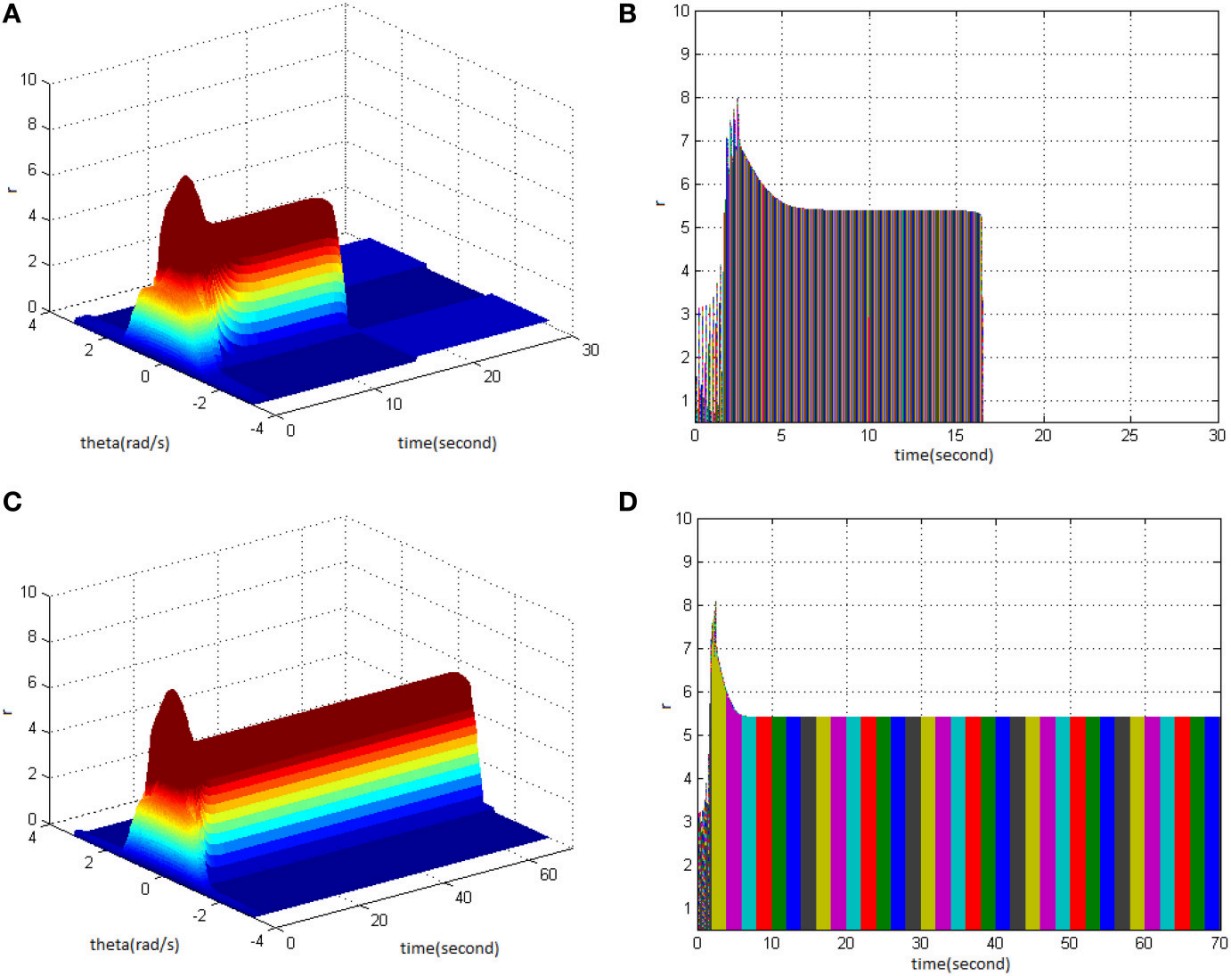
**Critical value of TBS properties to produce long-term memory. (A)** Space-time plots of the firing activity with TBS properties of cycle 13, amplitude 1.9, and duty ration 50%. **(B)** The projection of **(A)** in plane time-*r*. **(C)** Space-time plots of the firing activity with TBS properties of cycle 13, amplitude 2, and duty ration 50%. **(D)** The projection of **(C)** in plane time-*r*.

**Figure 7:**
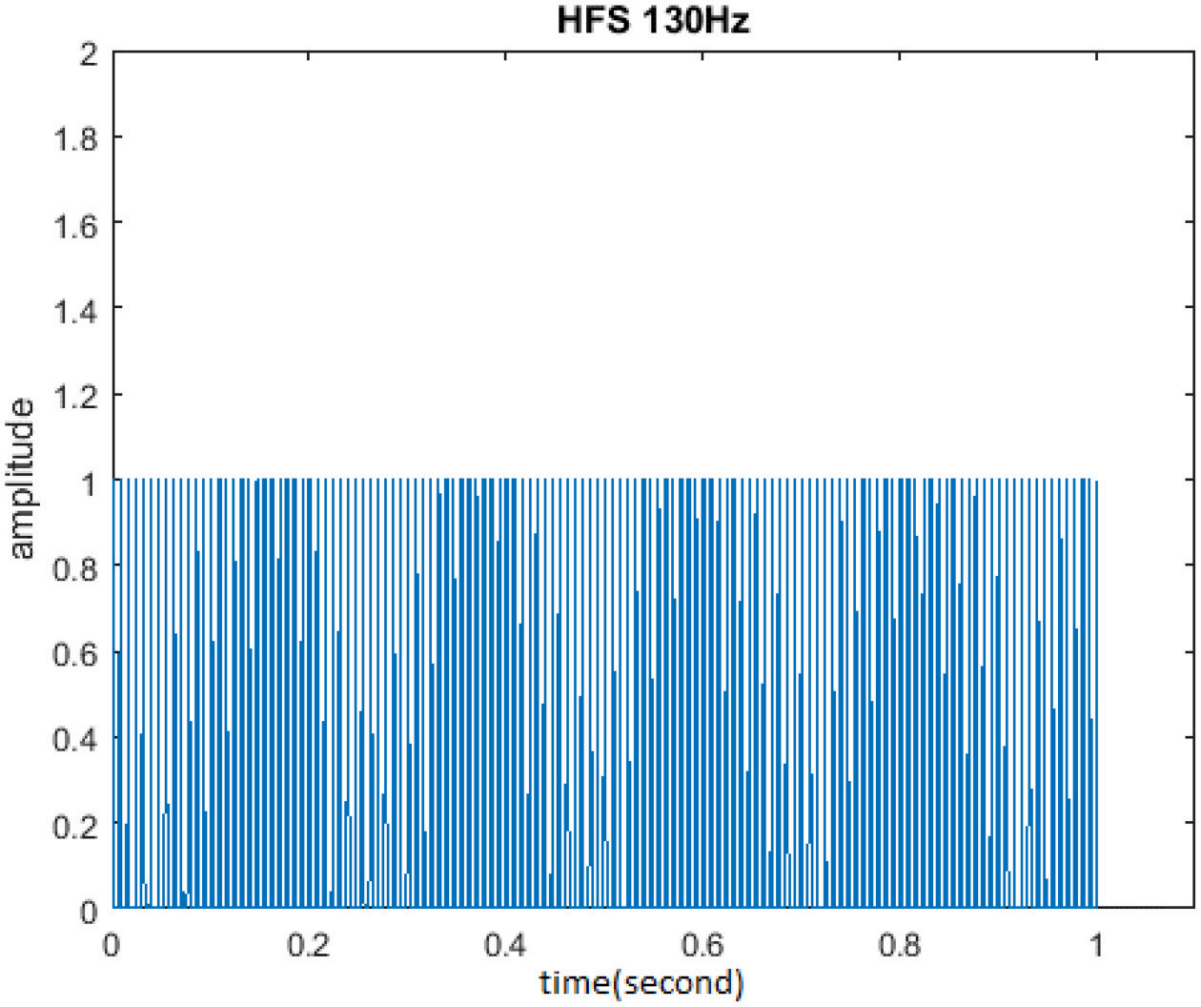
**Initial form of HFS**. HFS consisted of 130 pulses at 130 Hz in which cycle was 1, amplitude was 1, and duty ration was 50%.

**Figure 8:**
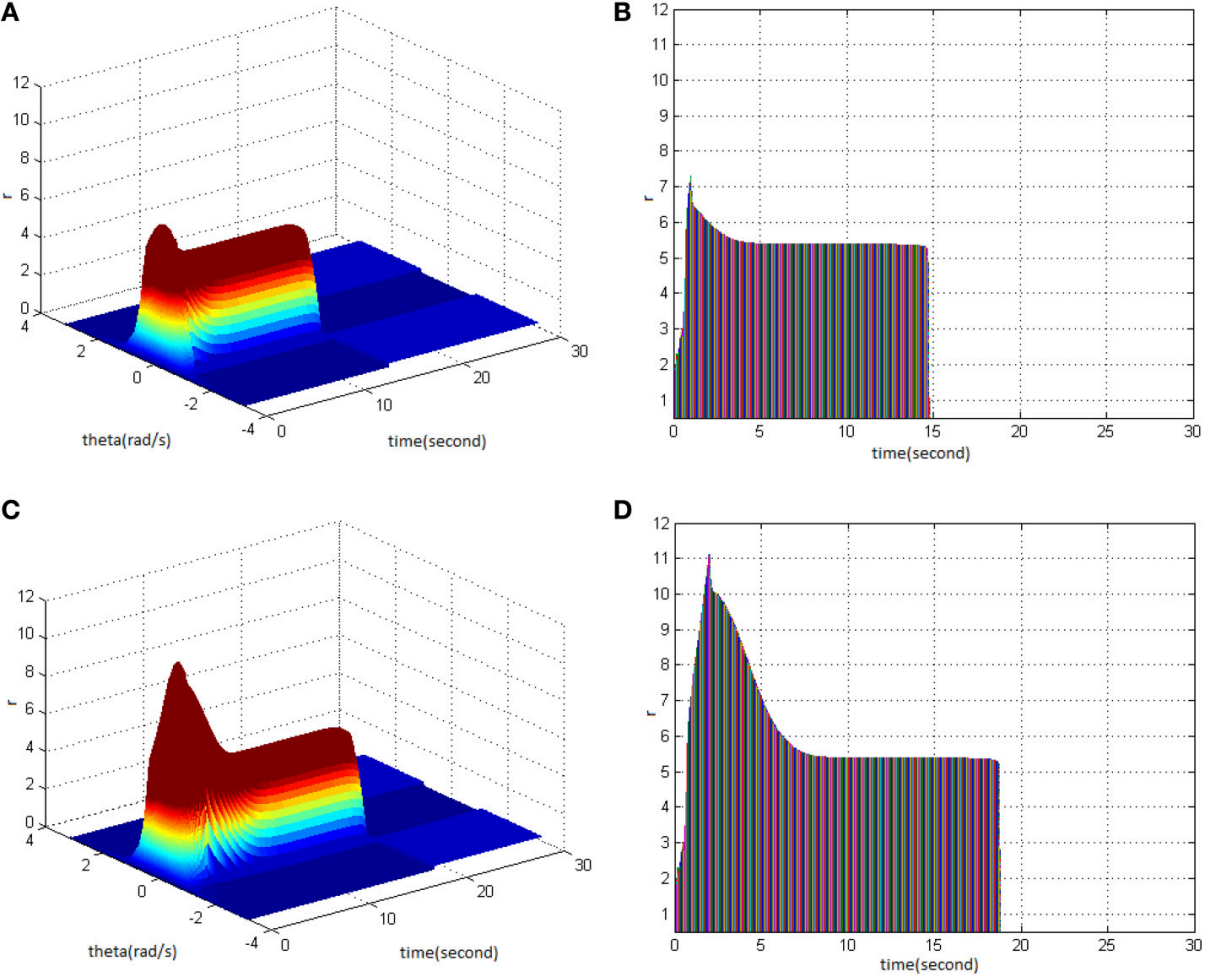
**Effect of HFS cycle on memory performance. (A)** Space-time plots of the firing activity with HFS properties of amplitude 1.2, duty ration 50%, and cycle 1. **(B)** The projection of **(A)** in plane time-*r*. **(C)** Space-time plots of the firing activity with HFS properties of amplitude 1.2, duty ration 50%, and cycle 2. **(D)** The projection of **(C)** in plane time-*r*.

**Figure 9:**
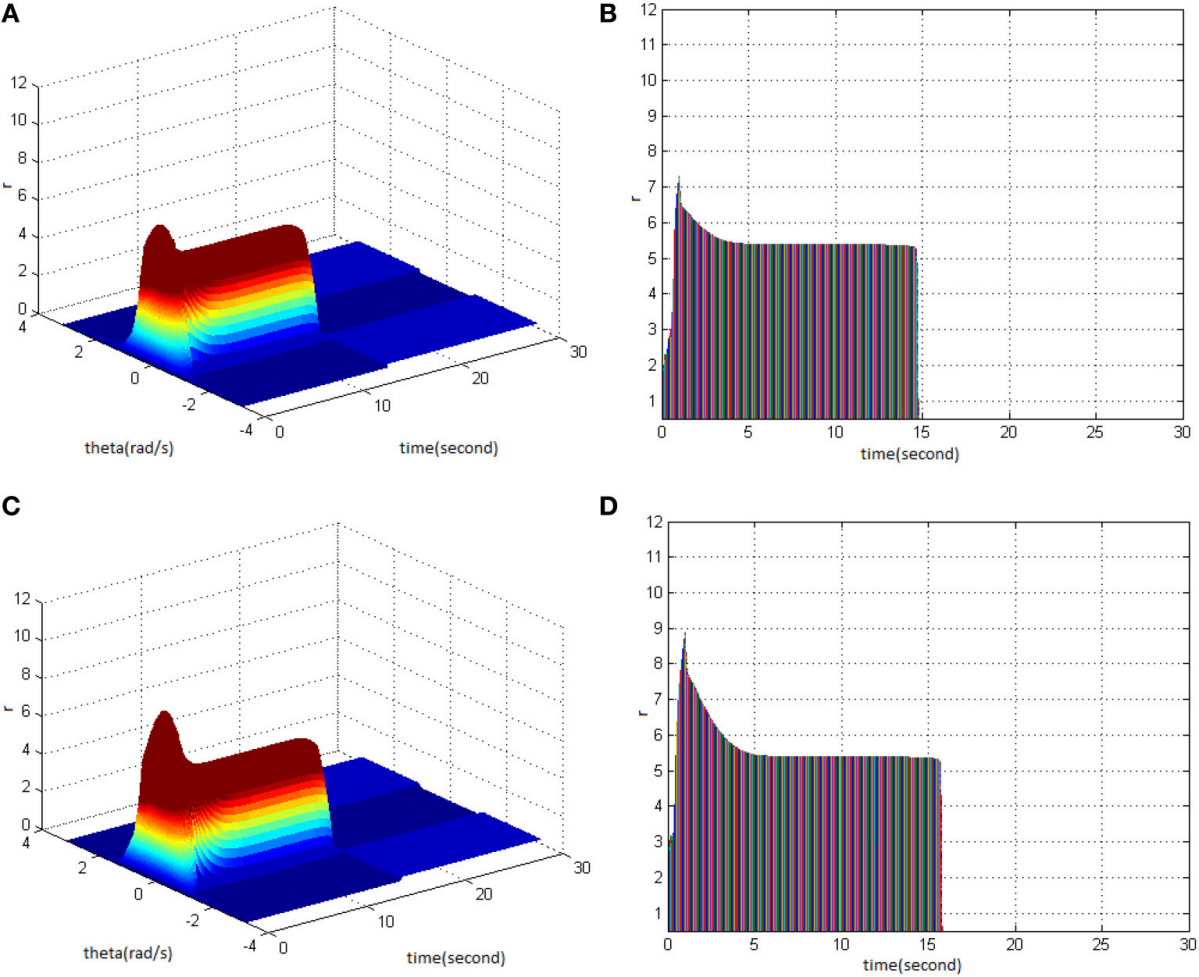
**Effect of HFS amplitude on memory performance. (A)** Space-time plots of the firing activity with HFS properties of cycle 1, duty ration 50%, and amplitude 1.2. **(B)** The projection of **(A)** in plane time-*r*. **(C)** Space-time plots of the firing activity with HFS properties of cycle 1, duty ration 50% and amplitude 1.5. **(D)** The projection of **(C)** in plane time-*r*.

**Figure 10:**
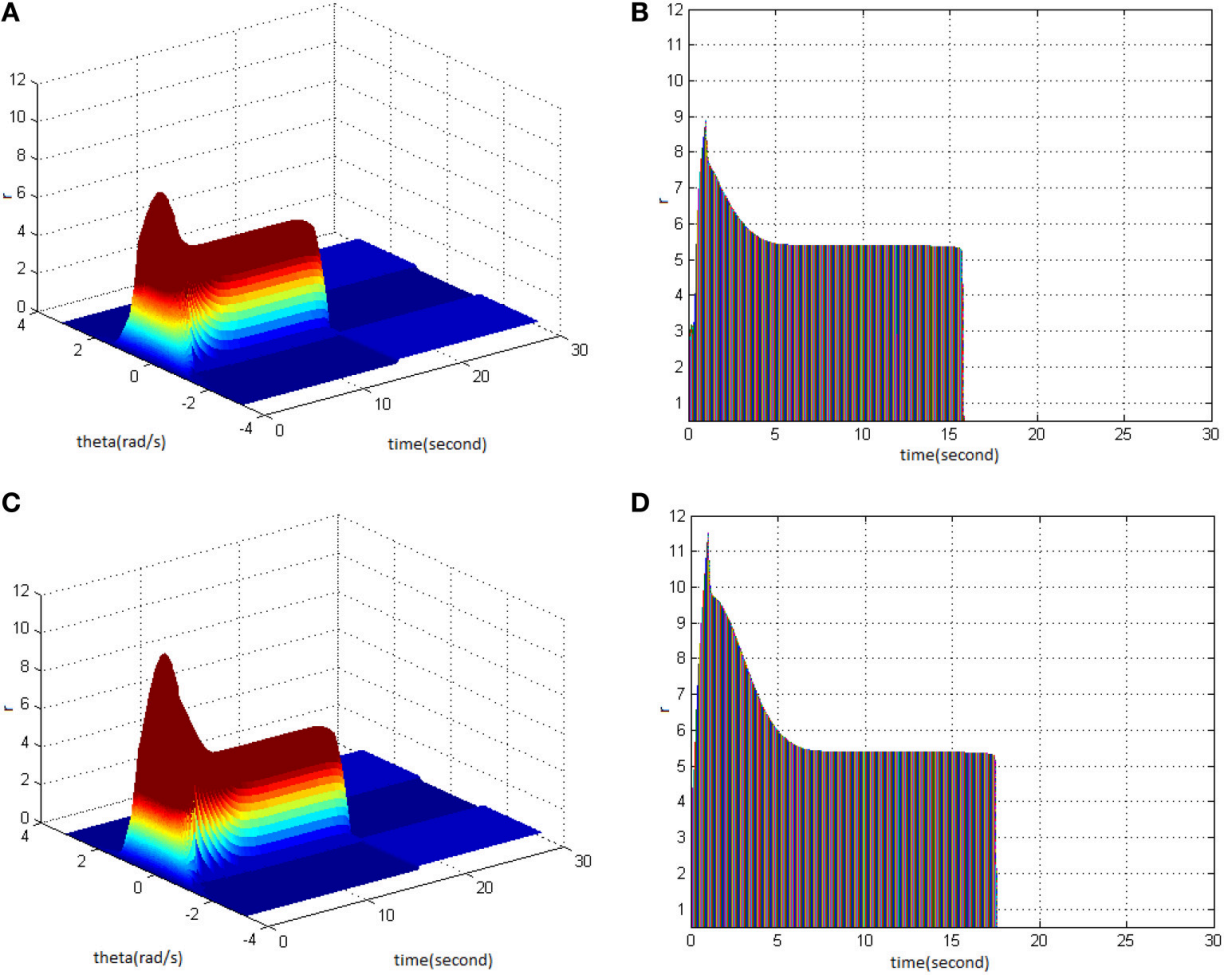
**Effect of HFS duty ration on memory performance. (A)** Space-time plots of the firing activity with HFS properties of cycle 1, amplitude 1.5, and duty ration 50%. **(B)** The projection of **(A)** in plane time-*r*. **(C)** Space-time plots of the firing activity with HFS properties of cycle 1, amplitude 1.5, and duty ration 70%. **(D)** The projection of **(C)** in plane time-*r*.

**Figure 11:**
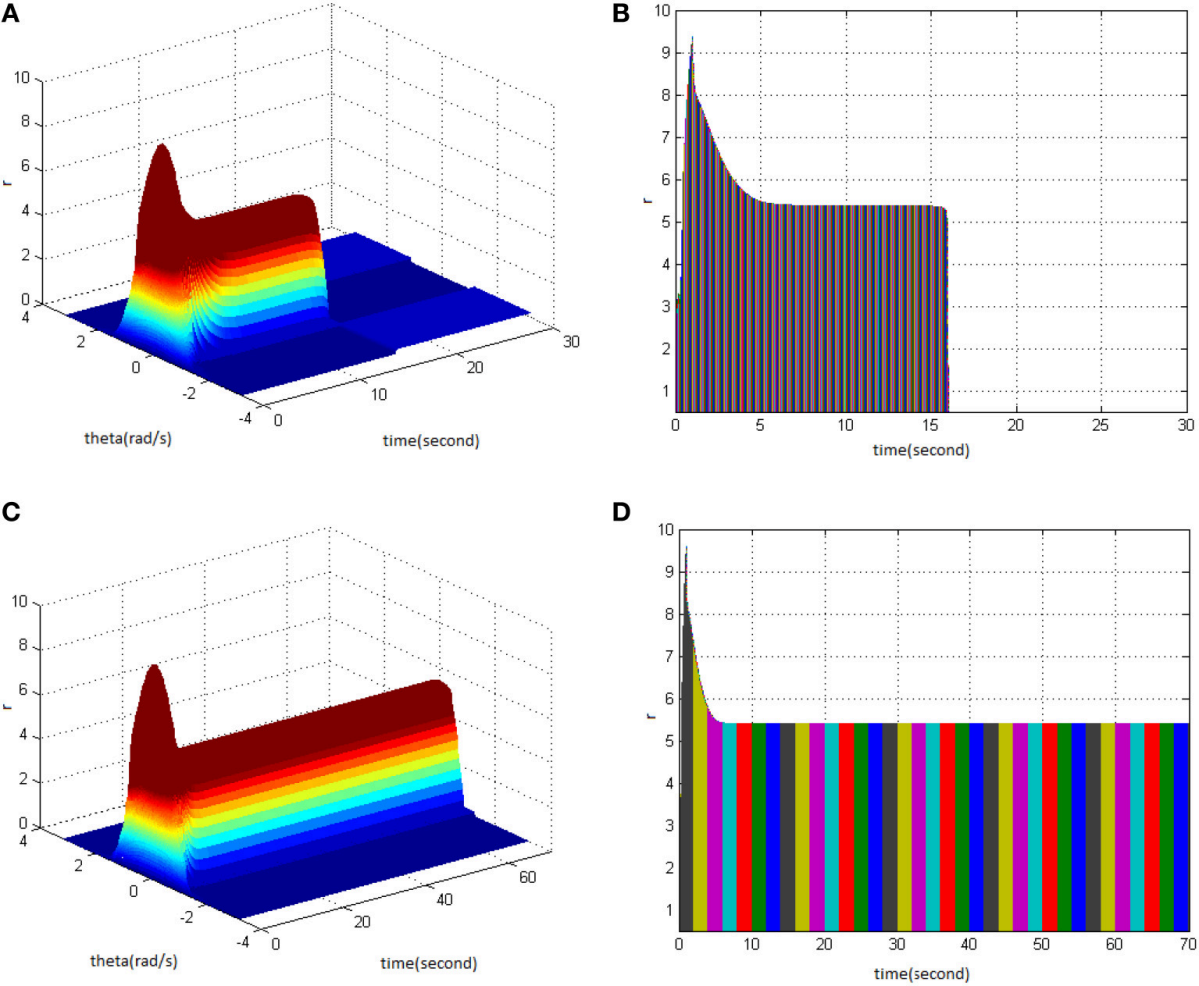
**HFS properties to produce long-term memory. (A)** Space-time plots of the firing activity with HFS properties of cycle 1, amplitude 1.7, duty ration 50%. **(B)** The projection of **(A)** in plane time-*r*. **(C)** Space-time plots of the firing activity with HFS properties of cycle 1, amplitude 1.8, and duty ration 50%. **(D)** The projection of **(C)** in plane time-*r*.

**Figure 12:**
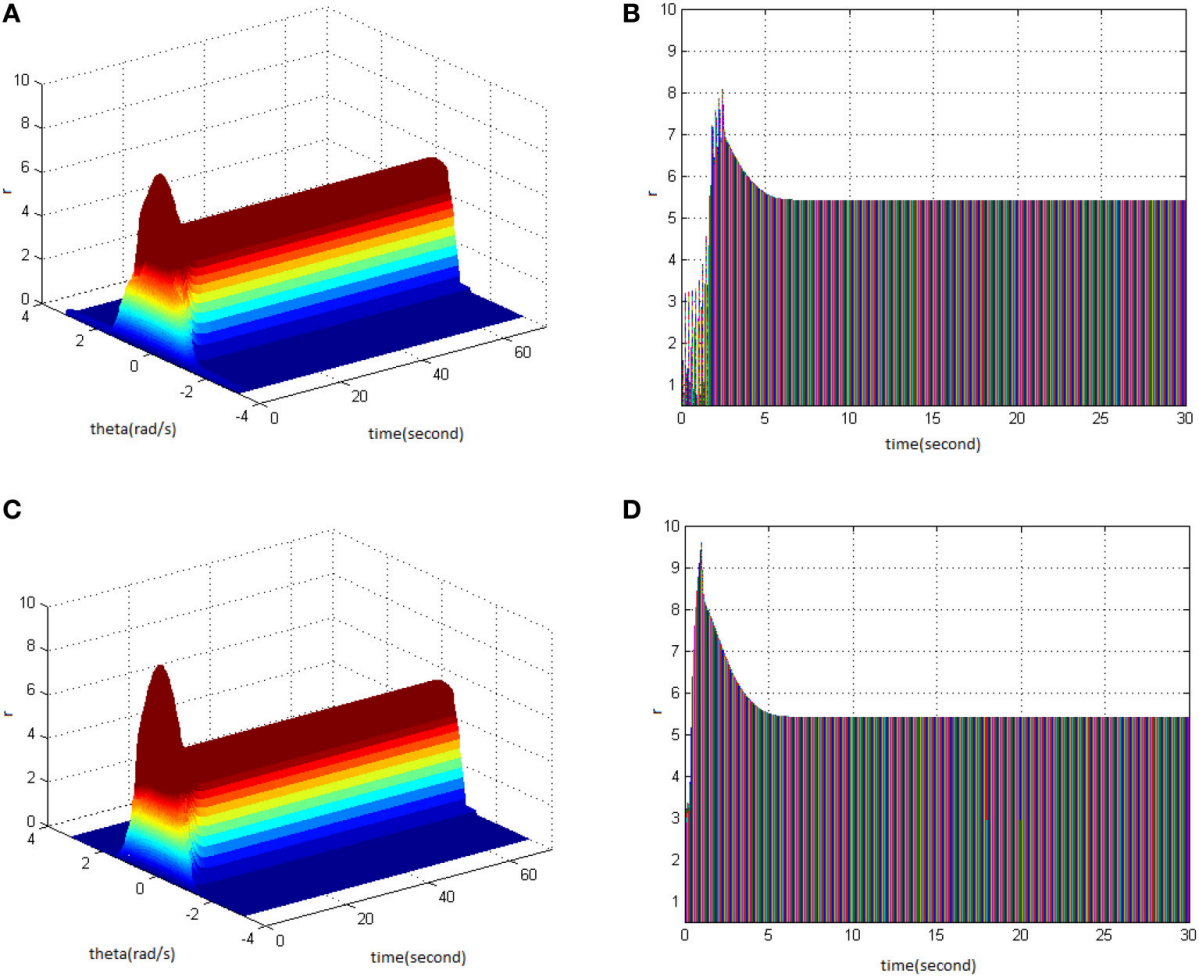
**Long-term memory induced by TBS and HFS with the identical pulse number. (A)** Space-time plots of the firing activity with TBS properties of cycle 13, amplitude 2, duty ration 50%. **(B)** The projection of **(A)** in plane time-*r*. **(C)** Space-time plots of the firing activity with HFS properties of cycle 1, amplitude 1.8, and duty ration 50%. **(D)** The projection of **(C)** in plane time-*r*.

First, TBS was set as amplitude 1.8 and duty ration 50%. Comparing the differences in network activities under the circumstances of stimulus cycle 10 and 30, the impact of stimulus cycle on memory performance could be revealed. Three-dimensional maps of the network activity with memory field θ and time *t* are shown in Figures 2A,C, in which Figure 2A shows cycle 10 and Figure 2C shows cycle 30. The duration of network activities reflects memory maintaining time. It is shown that with the increase of stimulus cycle, the maximum firing rate of network activities grew and memory maintaining time delayed. In order to gain insight into the relation between the increase, decrease and sustained process of firing rate and the time, the projection of Figures 2A,C in the plane time-*r* are shown in Figures 2B,D. This clearly demonstrates that the maximum of neuron firing rate increased from 6 (Figure 2A) to 10 (Figure 2B), and the period of network activities with sustained high firing patterns extended to 6 s. In Figures 2–6, 8–12 mentioned in the later paper, the right figures (Figures 2B,D) are the projections of the left figures in the plane time-*r*, thus special instructions were not mentioned afterwards.

Then, TBS was set as cycle 30, duty ration 50%. Comparing the differences in network activities under the circumstances of stimulus amplitude 1.6 (Figure 3A) and 1.8 (Figure 3C), the impact of stimulus amplitude on memory performance could be revealed. It is shown that with the increase of stimulus amplitude, the maximum firing rate of network activities grew and memory maintaining time delayed. The right side of Figure 3 shows that the maximum of neuron firing rate increased from 9 (Figure 3B) to 10 (Figure 3D), and the period of network activities with sustained high firing patterns extended from 21 to 22 s.

Lastly, TBS was set as cycle 30 and amplitude 1.4. Comparing the difference in network activities under the circumstances of stimulus duty ration 65% (Figure 4A) and 80% (Figure 4C) revealed the impacts of stimulus amplitude on memory performance. It is shown that the duty ration changed firing pattern of network activities for a short period. This occurred after network had reached the maximum firing rate, and memory maintaining time delayed. Figure 4D clearly shows that the peak of neuron firing rate exceeded 12 and sustained high firing rate for 2 s after reaching the peak; memory time exceeded 4 s compared to Figure 4B.

Study shows that adjusting the properties (cycle, amplitude, and duty ration) of TBS would change the network firing pattern, so as to impact to some extent on the memory time in the model. Therefore, adjusting the comprehensive effect of the three kinds of stimuli properties of TBS could transform working memory to long-term memory. Stimulus properties were first set as cycle 30, amplitude 1.8, and duty ration 50% to generate a network firing pattern (Figure 5A) and its corresponding projection in plane time-*r* (Figure 5B). The memory time was 21 s, indicating that it belongs to working memory. Then, setting stimulus properties as cycle 30, amplitude 1.9, and duty ration 50% generated network firing pattern (Figure 5C) and its corresponding projection in plane time-*r* (Figure 5D). The memory time was 70 s, and it met the requirements of long-term memory for time (more than 60 s). That is to say that, when the stimulus was set as cycle 30 and duty ration 50%, amplitude 1.9 was the critical value to produce long-term memory for the model. Therefore, analyzing the comprehensive effect of three properties of TBS on the model made the model transform from working memory to long-term memory.

The difference in stimulus properties between TBS and HFS during the process of long-term memory formation was compared further with the pulse number of TBS set to be in agreement with that of HFS in the following part, i.e., cycle 13 for TBS. Moreover, when the stimulus was set as amplitude 2 and duty ration 50%, long-term memory was produced (Figures 6C,D). However, when TBS was set as cycle 13 (130 pulses), amplitude 1.9, and duty ration 50%, long-term memory was not produced (Figures 6A,B). This means that when the stimulus was set as cycle 13 and duty ration 50%, amplitude 2 was the critical value to produce long-term memory for the model. In comparison with the results in Figures 5C,D, it could be deduced that using the same stimulus duty ration, with decreasing cycle requires higher amplitude to activate the working memory model so as to produce long-term memory.

The initial form of another stimulus HFS studied in this paper is shown in Figure 7. HFS consists of 1 s burst of equally spaced pulses at 130 Hz, in which cycle was 1. One second was defined as a cycle when amplitude was 1 and duty ration was 20%. The research process was similar to that of TBS and the unchanged parameters include: stimulus frequency 130 Hz and pulse number of each cycle 130. Cycle, amplitude, and duty ration should be adjusted so as to analyze the impact of these three stimuli properties on the memory model.

Firstly, HFS was set as amplitude 1.2 and duty ration 50%. Comparing the difference between network activities under the circumstances of stimulus cycle 1 (Figure 8A) and 2 (Figure 8C) revealed the impact of stimulus cycle on memory performance. It is shown that with increasing cycle, the maximum firing rate of network activities increased, and the memory time delayed. Figures 8B,D clearly shows that the maximum firing rate increased from 7 to 11, and memory time extended from 15 to 19s.

Secondly, HFS was set as cycle 1 and duty ration 50%. Evaluating the difference of network activities under the circumstances of stimulus amplitude 1.2 (Figure 9A) and 1.5 (Figure 9C) revealed the impact of stimulus amplitude on memory performance. It is shown that the maximum firing rate of network activities grew with the increase of stimulus amplitude. Figures 9B,D clearly shows that the maximum firing rate increased from 7 to 9, and memory time extended from 15 to 16 s.

Lastly, HFS was set as cycle 1 and amplitude 1.5. Comparing the difference in network activities under the circumstances of stimulus duty ration 50% (Figure 10A) and 70% (Figure 10C) revealed the impact of stimulus duty ration on memory performance. It is shown that the maximum firing rate of network activities increased. Figures 10B,D clearly shows that the maximum firing rate increased from 9 to 11, and memory time extended from 16 to 17 s.

From the study, it could be seen that adjusting the properties (cycle, amplitude, and duty ration) of HFS would change the network firing pattern, so as to impact to some extent on the memory time in the model. Therefore, adjusting the comprehensive effect of the three kinds of stimuli properties of HFS could transform working memory into long-term memory. First, stimulus properties were set as cycle 1, amplitude 1.7, and duty ration 50% to generate network firing pattern (Figure 11A) and its corresponding projection in plane time-*r* (Figure 11B). The memory time was 16 s, indicating that it belongs to the working memory. Then, the stimulus properties were set as cycle 1, amplitude 1.8, and duty ration 50%, to generate network firing pattern (Figure 11C) and its corresponding projection in plane time-*r* (Figure 11D). The memory time was 70 s and it met the requirements of long-term memory for time. That is to say that when the stimulus was set as cycle 1 and duty ration 50%, amplitude 1.8 was the critical value to produce long-term memory for the model. Therefore, analyzing the comprehensive effect of three properties of HFS on the model made the model transform from working memory to long-term memory.

The findings showed that the appropriate TBS and HFS could activate the working memory model in order to produce the long-term memory. The following figure shows the critical values of stimuli properties that can induce long-term memory when TBS and HFS have the same pulse number. Figure 12A is a network firing pattern under TBS, in which stimulus cycle was 13 (pulses 130), amplitude 2, and duty ration 50%; Figure 12C is a network firing pattern under HFS, in which stimulus cycle was 1 (pulses 130), amplitude 1.8, and duty ration 50%. Comparing the projection patterns (Figures 12B,D) of the first 30 s network activities under the two types of stimuli showed the differences in neuronal responses caused by them. When HFS and TBS were of the identical pulse number and duty ration, HFS could activate the network to give higher firing rate with less time and smaller amplitude. This produced long-term memory in the model. During the process of long-term memory formation, HFS presumably had lower stimulation cost and induced long-term memory more easily when compared to TBS.

## Conclusion

In this work, the specific stimuli were used in the working memory model to produce long-term memory. Improved C–W model with Ca^2+^ subsystem-induced bi-stability was adopted, and the changes in memory mode were studied under the adjustment of the properties (cycle, amplitude, and duty ration) of the two types of stimuli, TBS and HFS. It revealed the circumstances under which the working memory could evolve into long-term memory. The research shows that changing the network firing pattern has an impact to some extent on the memory maintaining time in the model. In other words, increasing the cycle, amplitude, and duty ration would increase the firing rate of network activities and make memory last longer.

The comprehensive effect of the three stimuli properties of TBS and HFS on memory performance was analyzed. And it showed that both stimuli (TBS, HFS) could activate the model to achieve the transformation from working memory to long-term memory. Specifically, TBS was set as cycle 13, duty ration 50%, and when amplitude increased to 2, working memory model was activated to produce long-term memory; HFS was set as cycle 1, duty ration 50%, and when amplitude increased to 1.8, the working memory model was induced to produce long-term memory. Comparing the critical values of stimuli properties for the two types of stimuli when they could induce long-term memory, it showed that the two types of stimuli were of the same pulse number and duty ration but the amplitude of HFS was smaller. As for the performance of inducing long-term memory, HFS had a lower stimulation cost. This means that it could more easily activate the working memory model to produce long-term memory. Two LTP induced protocols were adopted to successfully produce the long-term memory in the working memory model. They helped in understanding the relation between the working memory and the long-term memory. Furthermore, they provided theoretical basis for the study of neural dynamics mechanism of the long-term memory formation in the future and developing neural chips of long-term memory.

## Author contributions

YZ make substantial contributions to the research design, modeling, simulation and article drafting; RW make important contributions to the research concept and final proofreading; YW make some contributions in data analysis.

### Conflict of interest statement

The authors declare that the research was conducted in the absence of any commercial or financial relationships that could be construed as a potential conflict of interest.
